# *Endlicheria bracteolata* (Meisn.) Essential Oil as a Weapon Against *Leishmania amazonensis*: In Vitro Assay

**DOI:** 10.3390/molecules24142525

**Published:** 2019-07-10

**Authors:** Mariana Margatto Rottini, Ana Claudia Fernandes Amaral, José Luiz Pinto Ferreira, Edinilze Souza Coelho Oliveira, Jefferson Rocha de Andrade Silva, Noemi Nosomi Taniwaki, Arith Ramos dos Santos, Fernando Almeida-Souza, Celeste da Silva Freitas de Souza, Kátia da Silva Calabrese

**Affiliations:** 1 Laboratório de Imunomodulação e Protozoologia, Instituto Oswaldo Cruz, Fundação Oswaldo Cruz, Rio de Janeiro 21045-900, Brazil; 2 Laboratório de Plantas Medicinais e Derivados (PN1), Farmanguinhos, FIOCRUZ, Rio de Janeiro 21041-250, Brazil; 3 Laboratório de Cromatografia, Departamento de Química, UFAM, Manaus 69080-900, Amazonas, Brazil; 4 Núcleo de Microscopia Eletrônica, Instituto Adolfo Lutz, São Paulo 01246-000, Brazil; 5 Pós-graduação em Ciência Animal, Universidade Estadual do Maranhão, São Luís 65055-310, Maranhão, Brazil

**Keywords:** essential oil, leishmanicidal activity, cytotoxicity, transmission electron microscopy

## Abstract

The difficulties encountered and the numerous side effects present in the treatment of cutaneous leishmaniasis have encouraged the research for new compounds that can complement or replace existing treatment. The growing scientific interest in the study of plants, which are already used in folk remedies, has led our group to test *Endlicheria bracteolata* essential oil against *Leishmania amazonensis*. Several species of the *Lauraceae* family, or their compounds, have relevant antiprotozoal activities Therefore, the biological potential on *L. amazonensis* forms from the essential oil of *Endlicheria bracteolata* leaves was verified for the first time in that work. The antileishmanial activity was evaluated against promastigotes and intracellular amastigotes, and cytotoxicity were performed with J774.G8, which were incubated with different concentrations of *E. bracteolata* essential oil. Transmission electron microscopy and flow cytometry were performed with *E. bracteolata* essential oil IC_50_. Promastigote forms showed *E. bracteolata* essential oil IC_50_ of 7.945 ± 1.285 µg/mL (24 h) and 6.186 ± 1.226 µg/mL (48 h), while for intracellular amastigote forms it was 3.546 ± 1.184 µg/mL (24 h). The CC_50_ was 15.14 ± 0.090 µg/mL showing that *E. bracteolata* essential oil is less toxic to macrophages than to parasites. Transmission electron microscopy showed that *E. bracteolata* essential oil treatment is capable of inducing mitochondrial damage to promastigote and intracellular amastigote forms, while flow cytometry showed ΔѰm disruption in treated parasites. These results could bring about new possibilities to develop products based on *E. bracteolata* essential oil to treat cutaneous leishmaniasis, especially for people who cannot receive the conventional therapy.

## 1. Introduction

Leishmaniasis comprises a group of tropical diseases caused by obligate intramacrophage protozoa, which are transmitted by the bite of a female phlebotomine [1]. Leishmaniasis has several diverse clinical manifestations that include the cutaneous, mucosal and visceral forms [2], which present considerable rates of morbidity and mortality [3]. In Brazil, about 21,000 cases of cutaneous leishmaniasis/year were recorded from 2010 to 2014 and the detection rate is on average 13 cases/100,000 inhabitants. Currently the annual incidence for cutaneous leishmaniasis (CL) is one million people worldwide in the last five years [4,5].

Pentavalent antimonials have been used for decades to treat leishmaniasis. They are considered the drug of first choice, despite their severe side effects including headache, arthralgia, nausea, vomiting, pancreatitis and heart and kidney problems, besides the long and painful parenteral treatment. Amphotericin B, a second-choice drug, also has many side effects [3,6,7]. Thus, there is an urgent need to develop new drugs that are more effective and present minimal side effects.

For many years, natural products such as plants or their derivatives have been exploited and used in folk medicine for the control and treatment of various diseases. In Brazil although a large number of medicinal plants have been used popularly, there is little scientific evidence supporting the effectiveness of these alternative treatments. Recently, medicinal plants have become targets of intense research into their potential activity and their pharmacological effects [8,9,10]. Various studies have shown that essential oils have promising results in pharmacological trails against *Leishmania* species [11,12,13]. In addition, studies with essential oils from Brazilian Amazon flora have demonstrated promising potential against *Leishmania* species. Some of the species that have been tested: *Licaria canella*, whose main constituent is the aromatic ester benzyl benzoate (73.0%), which demonstrated an IC_50_ of 19 µg/mL [14]. The antileishmanial activity of *L. canella* essential oil was moderated in *L. amazonensis* promastigotes and it was observed low cytotoxicity of these essential oils on uninfected mice peritoneal macrophages, even when used in quantity twice as large as his IC_50_ and comparable to pentamidine, the reference drug. *Annona foetida*, whose IC_50_ is 16.2 µg/mL, and main active ingredient is the sesquiterpene bicyclogermacrene (35.12%) [15] demonstrated an antileishmanial activity in *L. amazonensis* and also low cytotoxicity to mice peritoneal macrophages of 6%. Also, the essential oil of two *Piper* species, i.e. *P. demeraranum*, which major components are limonene (19.3%) and β-elemene (33.1%) and *P. duckei*, which major components are germacrene D (14.7%) and trans-caryophyllene (27.1%) were assayed against *L.* amazonensis promastigotes and amastigotes forms. These oils inhibited the growth of amastigote forms of *L. amazonensis* more than promastigotes, not being toxic to the uninfected mice peritoneal macrophages cells [16].

The species *Endlicheria bracteolata* (Meisn.) C.K. Allen (Lauraceae family) are found in the Amazon regions of the Amapá, Pará, Amazonas and Acre states, where it is known as laurel. A folk preparation is made with macerated leaves, soaked in cold water and squeezed into a decoction. No biological studies of this species were found in the literature. Therefore, the aim of the present paper is to study the in vitro activity of the *E. bracteolata* essential oils against *Leishmania amazonensis*.

## 2. Results

### 2.1. Gas Chromatography/Mass Spectrometry (GC/MS) Analysis

Hydrodistillation of *Endlicheria bracteolata* leaves yielded 1.01% (*w*/*w*) of colorless oil with a strong odor. The analysis of the oil chromatogram obtained by GC-MS resulted in the identification of 31 components (Table 1), 85.8% of which are sesquiterpenes. The main constituent was an oxygenated sesquiterpene guaiol (46.4%).

### 2.2. Antileishmanial Activity Against Leishmania amazonensis Promastigote Forms and Cytotoxicity of E. bracteolata Essential Oil

**Figure 1:**
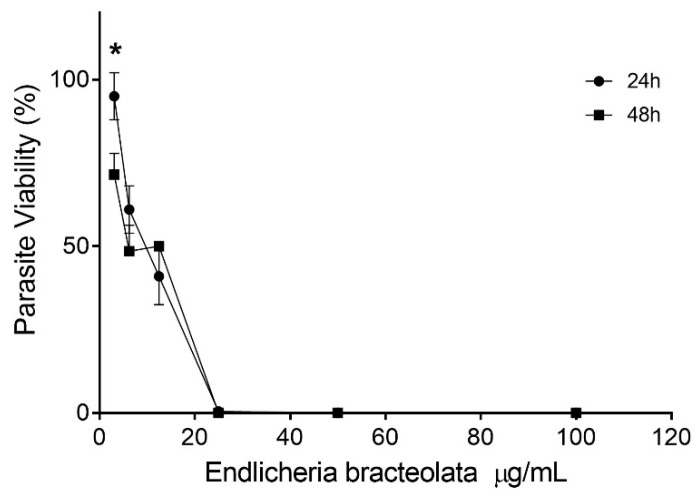
Effects of *Endlicheria bracteolata* essential oil on growth inhibition of *Leishmania amazonensis* promastigote forms. Data represents the mean ± error of three independent experiments carried out in triplicate. * *p* = 0.0168 when compared concentration treatment at different times by two-way ANOVA and Bonferroni’s multiple comparisons test.

*L amazonensis* promastigote forms were incubated with *E. bracteolata* essential oil for 24 and 48 h. The results showed that this essential oil presented a concentration-dependent activity and a discreet time-dependent activity only at the lowest concentration (3.125 µg/mL) (Figure 1). *E. bracteolata* essential oil was able to inhibit 50% of parasite growth (IC_50_) in 24 h of treatment at the concentration of 7.945 ± 1.285 µg/mL and the IC_50_ in 48 h was 6.186 ± 1.226 µg/mL. There was no statistical difference between the IC_50_ values of these treatment times. The 50% cytotoxic concentration (CC_50_) on J774.G8 macrophages was 15.14 ± 0.09 µg/mL, showing that *E. bracteolata* essential oil was more toxic to the parasites than the J774.G8 cells (Table 2).

### 2.3. Antileishmanial Activity Against L. amazonensis Intracellular Amastigotes Forms of E. bracteolata Essential Oil

**Figure 2:**
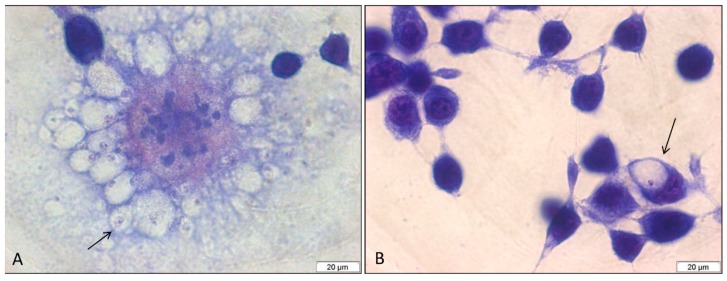
Light microscopy of *Leishmania amazonensis* intracellular amastigotes in J774.G8 macrophages. (**A**) *L. amazonensis* infected non-treated cells showing numerous internalized amastigotes (arrow). (**B**) *L. amazonensis* infected cells treated with 7.93 µg/mL of *Endlicheria bracteolata* essential oil against showing few amastigotes (arrow). Giemsa.

**Figure 3:**
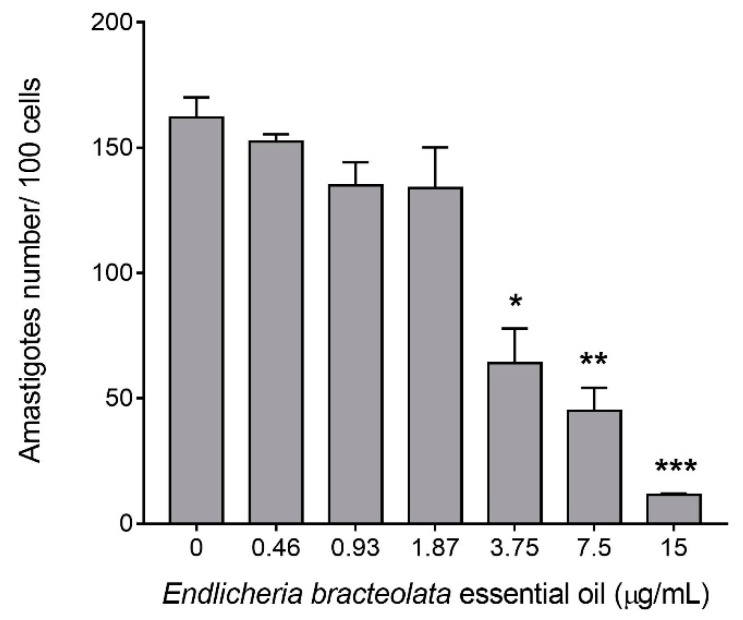
Intracellular amastigotes number of J774.G8 macrophages treated with *Endlicheria bracteolata* essential oil for 24 h. Each column represents the mean number of intracellular amastigotes in cell cultures treated with different concentrations of *E.* *bracteolata* essential oil. The value represents the number of amastigotes counted in 100 cells in three independent experiments carried out in duplicate. * *p* < 0.05; ** *p* < 0.01; *** *p* < 0.001 when compared to untreated cells by Kruskal-Wallis and Dunn’s multiple comparisons test.

The antileishmanial activity of *E. bracteolata* essential oil against *L. amazonensis* intracellular amastigotes was performed to evaluate the effects of this oil against parasites present inside macrophages. Analysis of cells by light microscopy showed that 99% of the non-treated control cells were infected with an average of 3.33 amastigotes per cell. The cells infected and treated with 7.93 µg/mL of *E. bracteolata* essential oil, which corresponds to the IC_50_ value for promastigotes, showed an infection rate of 62% with an average of 1.04 amastigotes per cell (Figure 2). However, when infected cells were treated with 3.75, 7.5 and 15 µg/mL of *E. bracteolata* essential oil, the number of intracellular amastigotes observed inside the cells decreased drastically (Figure 3). Moreover, in this assay it was possible to calculate the IC_50_ value of *E. bracteolata* essential oil for the intracellular amastigotes, demonstrating that this essential oil is able to inhibit 50% of amastigotes with 3.546 ± 1.184 µg/mL (Table 2). Selectivity index showed that *E. bracteolata* essential oil is 4.27-fold more active to intracellular amastigote than to J774.G8 macrophage cells.

### 2.4. Transmission Electron Microscopy of the Promastigote Forms

**Figure 4:**
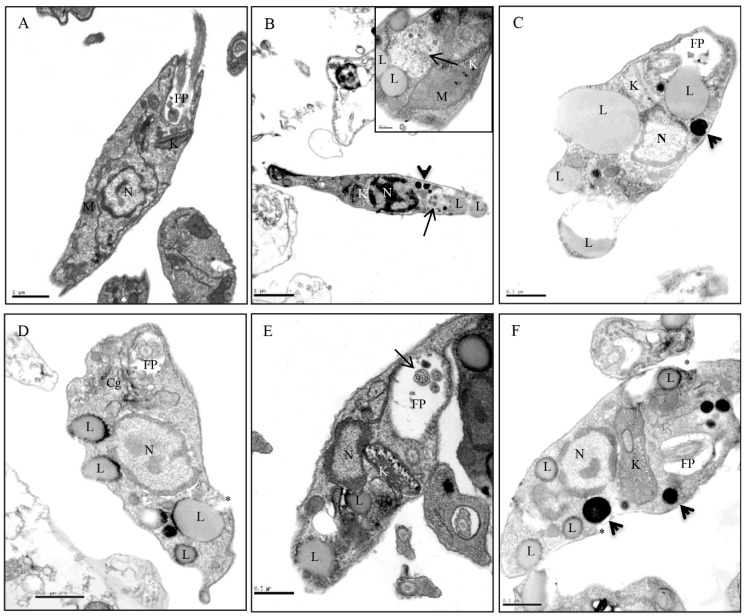
Transmission electron microscopy of *Leishmania amazonensis* promastigote forms treated for different times with *Endlicheria bracteolata* (7.93 μg/mL). (**A**) Untreated parasites showing the characteristic structure of kinetoplastids (K), flagellar pocket (FP), and nucleus (N). (**B**) Promastigotes treated for 2 h showing electron-dense structures (arrowhead), in the box above note the presence of multivesicles (arrow), mitochondrial swelling (M) and presence of lipid droplets (L). (**C**) 4 h of treatment showing kinetoplast swelling (K), increased number of lipid droplets (L) and electron-dense structures (arrowhead). (**D**) 8 h of treatment showing large lipid droplets close to the plasma membrane (L) and discontinuity of the plasma membrane (asterisk). (**E**) 16 h of treatment showing kinetoplast swelling (K), lipid droplets (L) and multivesicles inside the flagellar pocket (FP) (arrow). (**F**) promastigotes after 24h of treatment showing increased volume of the kinetoplast (K), increased number of lipid droplets (L) and electron-dense structures (arrowheads).

The electron microscopy analyses of the untreated promastigote forms showed the parasite with an elongated body and all its organelles intact (Figure 4A). However, promastigotes treated with of *E. bracteolata* essential oil IC_50_, 7.93 μg/mL, showed several alterations. Promastigotes treated for 2 h showed electron-dense structures, vacuoles similar to autophagosome, lipid droplets and mitochondrial swelling (Figure 4B). After 4 and 8 h of treatment kinetoplast swelling, increased number of lipid droplets close to the plasma membrane and electron-dense structures were observed (Figure 4C,D, respectively). After 16 h of treatment, kinetoplast swelling, lipid droplets and multivesicles inside the flagellar pocket were observed (Figure 4E). After 24 hours of treatment, the parasites showed an increase in the volume of their kinetoplasts, the presence of lipid droplets and more of electron-dense structures of different sizes (Figure 4F).

### 2.5. Transmission Electron Microscopy of Intracellular Amastigote Forms

**Figure 5:**
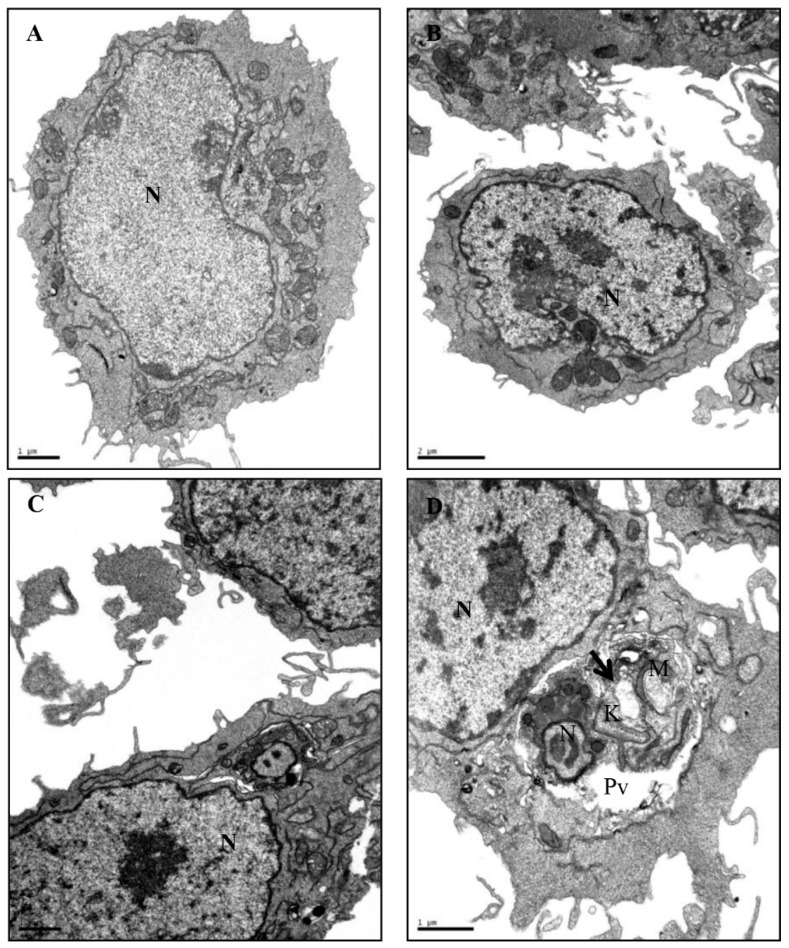
Ultrastructural effects of *Endlicheria bracteolata* essential oil on intracellular *Leishmania amazonensis* amastigotes and macrophage cells. (**A**) Untreated and uninfected macrophages showing typical morphology; (**B**) macrophages treated for 24h also showing typical morphology; (**C**) infected macrophages showing intact kinetoplast in the amastigote forms; (**D**) Infected and treated macrophages showing ultrastructural changes in the amastigotes such as the presence of vacuoles (arrows) in the cytoplasm and damage to their mitochondria (M). No changes were observed in the morphology of the host cell. N: *nucleus*; M: *mitochondria*; K: *kinetoplast*; Pv: *parasitophorous vacuole*.

The ultrastructural analysis of non-infected J774.G8 macrophages (both treated and non-treated cells) showed normal morphology and preservation of cytoplasmic membranes (Figure 5A,B, respectively). The analysis of cells infected for 24 h showed an intact structure of the parasite and host cell. A well-defined nuclear membrane and the presence of the intact kinetoplast could be clearly seen (Figure 5C). However, analysis of infected and *E. bracteolata* essential oil treated cells showed ultrastructural changes in the amastigotes inside the vacuoles in the cell cytoplasm. Mitochondria and kinetoplast swelling, discontinuity of plasma membrane and change of shape of the parasite were observed without change to the host cell morphology (Figure 5D).

### 2.6. Flow Cytometric Analysis

**Figure 6:**
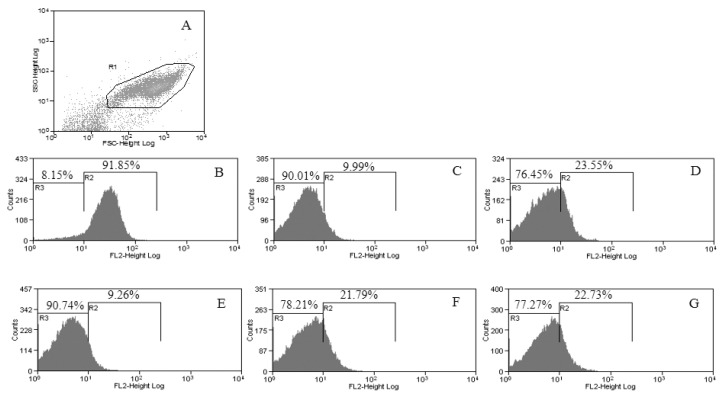
Flow cytometry of *Leishmania amazonensis* treated with 7.93 µg/mL of *Endlicheria bracteolata* essential oil to evaluate the mitochondrial membrane potential (ΔѰm). (**A**) Promastigotes captured in the gated region. (**B**–**G**) representative histograms of non-treated promastigotes incubated with TMRE showing ΔѰm intact in R2 and change of ΔѰm in R3. Promastigotes untreated (**B**) and treated for 2 h (**C**), 4 h (**D**), 8 h (**E**), 16 h (**F**), and 24 h (**G**).

A flow cytometric analysis was performed in order to monitor mitochondrial damage to the parasite when treated with 7.93 µg/mL of *E. bracteolata* essential oil and to assess the mitochondrial membrane potential (ΔѰm) with different times of treatment. For this analysis we used TMRE, a permeable positively charged dye that can detect the net negative charge across a healthy mitochondrion of viable cells [17]. The results of flow cytometry showed a disruption of the ΔѰm in parasites independently of *E. bracteolata* essential oil treatment time used. Parasites untreated and incubated with TMRE showed 91.85% of viable ΔѰm (Figure 6B). When we analysed the parasites treated for 2 h we noticed a disruption of 90.01% in the ΔѰm (Figure 6C). After 4 h of treatment 76.45% of the mitochondria were damaged (Figure 6D). Parasites treated for 8 h, showed 90.74% of mitochondrial damage (Figure 6E). After 16 h, the disruption of ΔѰm was 78.21% (Figure 6F) and 24 h after treatment the mitochondrial damage was 77.27% in the promastigotes forms treated with IC_50_ of *E. bracteolata* essential oil (Figure 6G).

## 3. Discussion

During the last decade, different essential oils and compounds isolated from plants began to be investigated and many of them have shown antileishmanial activity [10,11,18]. Recently, studies with species of the Brazilian Amazonian flora have attracted the attention of the scientific community to interesting and promising essential oils, such as *Annona foetida* [15]; *Licaria canella*, *Aniba canelilla* [14], *Piper aduncum* and *Piper demeraranum* [16]. The results of these studies showed antileishmanial activity against *L. amazonensis* promastigotes (IC_50_: 16.2–86 µg/mL) and amastigotes (IC_50_: 42.4–78 µg/mL). In the present work, we demonstrated that the essential oil of *E. bracteolata* has higher efficacy than previous studies, presenting IC_50_ values of 7.93 for promastigotes and 3.54 µg/mL for intracellular amastigotes (Table 2).

In previous works, mitochondrial alterations were reported when promastigote and amastigote forms were treated with some essential oils or with isolated compounds from medicinal plants. Mitochondrial swelling was observed in promastigote and amastigote forms treated with *Ocimum gratissimum* essential oil [19]; the presence of exocytic projections were described in the flagellar pocket of *L. amazonensis* treated with a citral compound [20] and cytoplasmic lipid accumulation was demonstrated in *L. amazonensis* treatment with the essential oil of *Lippia sidoides* [21]. Similar alterations were observed by us in this work using *L. amazonensis* treated with *E. bracteolata* essential oil. Besides these alterations, other changes were observed in the promastigotes treated with *E. bracteolata* essential oil such as, presence of vacuoles similar to autophagosome and electron dense structures of different sizes (Figure 4B–F).

Some authors have demonstrated alterations in parasites treated with essential oils, observing several modifications similar to those verified by us in the present study and they indicated that these alterations might be due to the inhibition of ergosterol synthesis [11,20,21]. Another hypothesis that may explain the effectiveness of *E. bracteolata* essential oil against *Leishmania* is the ability of the essential oil to cross the mitochondrial membrane of the parasite, due to its lipophilicity, leading to the death of the parasite. A study with two triterpenic acids from olive leaf extracts showed that these compounds are able to induce death of parasites of the genus *Leishmania* due to their ability to decrease the mitochondrial membrane potential (ΔѰm) [22]. Similar findings were reported in this work when promastigotes of *L. amazonensis* were treated with IC_50_ of *E. bracteolata* essential oil at different times. The use of TMRE, a dye that accumulates in the mitochondria of healthy cells, demonstrated that this essential oil is capable of crossing the plasma membrane causing a collapse of the mitochondrial membrane of the parasite. However, these damages were not time dependent, as the other changes found at different treatment times were.

The observation of vacuoles similar to autophagosome by transmission electron microscopy could be related to a degradation of damaged organelles induced by the essential oil treatment, instead of the mechanism of a cell death program, considering that autophagy in *Leishmania* remains controversial [23,24].

The intracellular amastigote forms were more susceptible to *E. bracteolata* essential oil than the promastigote forms (Figure 5B–D), corroborating the study of Ueda-Nakamura with eugenol-rich essential oil from *Ocimum gratissimum* [19]. This is probably because *E. bracteolata* essential oil increases the effectiveness of the macrophage microbicide response. These results could bring new hopes for the treatment of cutaneous leishmaniasis to people who cannot receive the conventional treatment such as pregnant women, children, and people with heart and nephropathic diseases; however, further experiments are needed. Thus, in this paper we demonstrated, in the light of our knowledge, for the first time, the *E. bracteolata* essential oil promising antileishmanial activity against *L. amazonensis*. More studies are necessary to elucidate this finding.

## 4. Materials and Methods

### 4.1. Plant Material

*E. bracteolata* leaves were collected in the Adolpho Ducke reserve, km 26 (latitude −2.908185, longitude −59.975457) on the Itacoatiara highway, Manaus, Amazonas State, Brazil. A voucher species was deposited at the Herbarium of INPA (Amazonas state, Brazil) under the number 179.096.

### 4.2. Extraction of the Essential Oil

Fresh and dried leaves of *E. bracteolata* (327 g) were ground and submitted to hydrodistillation (2 h) using a modified Clevenger-type apparatus. After each distillation, the oils were collected, dried using anhydrous sodium sulfate and placed in a centrifuge. The supernatant was removed and transferred to amber glass flasks and stored at 4 °C. The yields were calculated based on the weight of the plant material used.

### 4.3. Gas Chromatography Analysis

Chemical analysis was carried out by gas chromatography (GC) coupled to a mass spectrometer (MS), and by GC coupled to a flame ionization detector (FID) to identify the components of the oil. The gas chromatograph (GC) 6890N (Agilent Technologies, Santa Clara, CA, USA) was equipped with a mass detector 5973 Network (Agilen Technologies, Santa Clara, CA, USA), 7683B series injector (Agilent Technologies, Santa Clara, CA, USA) and a DB-5MS column of 30 m, 0.32 mm i.d. and 0:25 µm film thickness. The analysis was carried out with a temperature ranging from 40 °C to 290 °C with a run time of 67 min. Helium was used as the carrier gas, with a flow rate of 1 mL·min^−1^. The other parameters were: MS interface temperature: 280 °C; MS mode: EI; detector voltage: 70 eV; mass range: 40–700 u; scan speed: 150 u/s; interval: 0.50 s (2 Hz). The essential oil obtained from *E. bracteolata* leaves was also analyzed using a GC (HP 5890 GC system, Agilent Technologies) equipped with a DB-5 capillary column (30 m × 0.25 mm, 0.25 μm film thickness) and a FID detector. The oven temperature was programmed from 60 °C to 290 °C at a rate of 3 °C·min^−1^, then isothermal at 290 °C for 10 min, using H_2_ as the carrier gas (1.0 mL min^−1^). Injector and detector temperatures were 230 °C and 290 °C, respectively. The other parameters were: Injection volume: 1.0 μL. Carrier gas: He, linear velocity (ū): 14 cm/s Injection mode: splitless. The composition of the oil was determined by comparison of their retention indices and mass spectra with those reported in the literature [25] or presented in the Wiley data system library of the equipment. The retention indices were calculated for all volatile constituents using the n-alkane homologous series.

### 4.4. Cytotoxicity Assay

J774.G8 macrophages were plated at 1 × 10^5^ cells/well in 96-well microplates with Dubelco’s Modified Eagle medium (DMEM) (Sigma-Aldrich, St. Louis, MO, USA), supplemented with 10% inactivated fetal bovine serum (FBS) and incubated for 24 h at 37 °C in 5% CO_2_ to obtain a confluent monolayer. After 24 h, the medium was removed, and different concentrations of *E. bracteolata* essential oil (1.86–60 µg/mL) were added to each well. Positive control (with Amphotericin B diluted from 4 to 0.12 μg/mL) and negative controls (without drug) were included in each experiment. Twenty-four hours later the cells were washed in culture medium and a medium containing 100 µg/mL neutral red dye (Sigma, St. Louis, MO, USA) was added. After 3 h, the neutral red medium was discarded; cells were rinsed twice with warm (37 °C) phosphate-buffered saline (PBS) (pH 7.4) in order to remove the non-incorporated dye, and then 100 µl 50% ethanol and 1% acetic acid solution were added to each well to release the incorporated neutral red. The plates were then shaken for 10 min and the absorbance was measured in a spectrophotometer at 540 nm. Wells without cells were used as blank and wells only with cells were used as control. Cytotoxicity was calculated from the percentage of viable cells comparing treated cells with non-treated one. The 50% cytotoxic concentration (CC_50_) was determined by regression analysis using the GraphPad Prism 5.0 software (GraphPad Software Inc, San Diego, CA, USA). Each assay was carried out in triplicate in three independent experiments.

### 4.5. In Vitro Antileishmanial Activity of E. bracteolata Essential Oil Against L. amazonensis Promastigote Forms

*L. amazonensis* amastigotes (MHOM/BR/76/Ma-76) were isolated from BALB/c lesions and maintained as promastigotes at 26 °C in LIBHIT medium containing 10% inactivated FBS, 100 µg/mL streptomycin and 100 U/mL penicillin (Sigma-Aldrich, St. Louis, MO, USA). Promastigote forms in exponential growth were incubated for 24 and 48 h in the absence or in the presence of different concentrations, 3.12–100 µg/mL, of *E. bracteolata* essential oil. Incubation took place in a 96-wells plate, in a BOD incubator at 26 °C in LIBHIT medium using a parasite concentration of 10^6^ promastigotes/mL. After 24 and 48 h, the number of viable promastigotes was estimated by counting in a Neubauer chamber [26]. The concentration that inhibited parasite growth by 50% (IC_50_) was determined by linear regression analysis using the Graphpad Prism 5.0 software. Amphotericin B was used as the reference drug. Each assay was carried out in triplicate in three independent experiments. All experiments with animals were conducted in accordance with the guidelines for experimental procedures of Oswaldo Cruz Foundation (License LW 72/12).

### 4.6. In Vitro Antileishmanial Activity of E. bracteolata Essential Oil Against Intracellular Amastigote Forms

Female BALB/c mice were inoculated with 3 mL of sodium thioglycolate 3% and 72 hours after, peritoneal macrophages were harvested with PBS solution. The harvest cells were then centrifuged at 4000 rpm and plated at 1 × 10^4^ cells/well in 24-well culture plates with coverslips, containing DMEM supplemented with 10% inactivated FBS and incubated for 24 h at 37 °C under 5% CO_2_ atmosphere to evaluated if *E. bracteolata* essential oil was able to pass through the cell membrane and act on the intracellular amastigote forms. Non-adherent cells were washed out and the remaining macrophages were then infected with *L. amazonensis* promastigote forms, in the stationary growth phase using a ratio of 1:10. After 4 hours, cells were washed to remove extracellular parasites and treated with different concentrations of *E. bracteolata* essential oil (15 to 1.86 µg/mL) and then incubated at 35 °C for 24 h. Cells were then washed with PBS, fixed in methanol and stained with Giemsa. Infected and non-treated cells were used as controls. The percentage of infected macrophages was determined by counting 100 cells in duplicate. The survival index was determined by multiplying the percentage of infected macrophages by the mean number of parasites per infected cell. Amphotericin B was used as the reference drug. The 50% cytotoxic concentration (CC_50_) was determined by regression analysis using the Prism 5.0 software. Selectivity index were calculated from the ratio of CC_50_/IC_50_ of intracellular amastigote forms.

### 4.7. Transmission Electron Microscopy of Promastigotes and Intracellular Amastigotes

A transmission electron microscope was used to evaluate if *E. bracteolata* essential oil had caused any ultrastructural alterations to the *L. amazonensis* promastigote forms. Parasites were treated or not with *E. bracteolata* essential oil IC_50_ for different times (2, 4, 8, 16 and 24 h). After this period, the parasites were collected and processed as described elsewhere [27]. Ultrathin sections were stained with uranyl acetate and lead citrate, and examined in a Jeol JEM 1011 (JEOL, Tokyo, Japan) transmission electron microscope. The same assay was used to evaluate if *E. bracteolata* essential oil had caused any ultrastructural alterations in infected and non-infected cells and thus analyze its potential to kill intracellular amastigotes. Macrophages J774.G8 uninfected and infected with *L. amazonensis* were treated with *E. bracteolata* essential oil IC_50_ for 24 h at 37 °C and 5% CO_2_. Cells were then processed for transmission electron microscopy [26]. Each assay was carried out in duplicate in two independent experiments.

### 4.8. Flow Cytometric Assay

*L. amazonensis* promastigote forms (1 × 10^6^ parasites) were incubated at 26 °C, for 2, 4, 8, 16 and 24 h, with *E. bracteolata* essential oil IC_50_ (treated) or with culture medium (untreated). After these times, the parasites were incubated with 50 nM Tetramethylrhodamine Ethyl Ester (TMRE) (Molecular Probes, Carlsbad, USA). Flow cytometric assay was carried out to evaluate the mitochondrial membrane potential in a FACSCalibur flow cytometer (Becton Dickinson, CA, USA) as described in detail previously [26].

### 4.9. Statistical Analysis

Data were expressed as mean ± standard deviation. Group comparisons were performed with Kruskal–Wallis test and Dunn’s multiple comparison test, and analysis of concentration and time variables were performed with two-way ANOVA and Bonferroni’s multiple comparisons test. Statistical analysis was performed with GraphPad Prism 5.0 software.

## Figures and Tables

**Table 1:** Chemical composition of the *Endlicheria bracteolata* essential oil.

Compounds	Retention Index	Total Area (%)
**Monoterpenes**		
α-thujene	930	0.1
α-pinene	939	1.1
β-pinene	979	0.7
myrcene	990	0.1
α-phellandrene	1002	3.4
ρ-cymene	1024	2.0
limonene	1029	0.3
**Sesquiterpenes**		
α-copaene	1376	0.2
β-elemene	1390	0.5
(E)-caryophyllene	1419	3.8
β-gurjunene	1433	0.3
γ-elemene	1436	0.2
α-guaiene	1438	0.6
aromadendrene	1441	0.2
premnaspirodiene	1451	0.2
β-selinene	1490	1.1
δ-selinene	1492	0.1
viridiflorene	1496	0.1
Isodaucene	1499	0.5
α-muurolene	1500	0.2
α-bulnesene	1509	1.3
δ-cadinene	1523	0.1
selina-3,7(11)diene	1546	0.1
rosifoliol	1548	0.6
elemol	1549	2.6
occidentalol	1552	0.1
germacrene B	1561	3.0
guaiol	1600	46.4
10-epi- γ-eudesmol	1622	17.9
γ-eudesmol	1632	1.0
α-eudesmol	1653	1.2
α-cadinol	1654	0.1
bulnesol	1671	3.7
**Terpenoids Class**		
Monoterpenes		7.7
Hydrocarbon sesquiterpenes		12.5
Oxygenated sesquiterpenes		73.6
**Identified Compounds**		93.8

**Table 2:** Antileishmanial activity, cytotoxicity and selectivity index of *Endlicheria bracteolata* essential oil for 24 hours of treatment.

Compounds	*Leishmania amazonensis* IC_50_ (µg/mL)		J774.G8 CC_50_ (µg/mL)	SI
	Promastigote	Intracelular Amastigote		
***Endlicheria bracteolata* essential oil**	7.945 ± 1.285	3.546 ± 1.184	15.14 ± 0.090	4.27
Amphotericin B	1.521 ± 1.500	0.869 ± 0.774	19.97 ± 1.462	22.9

IC_50_: inhibitory concentration for 50% of parasites; CC_50_: cytotoxic concentration for 50% of cells; SI: selectivity index, obtained from ratio CC_50_/IC_50_ intracellular amastigote. Data represents mean ± standard deviation of three independent experiments carried out in triplicate.
